# Analysis of the Antioxidant and Antimicrobial Activity, Cytotoxic, and Anti-Migratory Properties of the Essential Oils Obtained from Cultivated Medicinal *Lamiaceae* Species

**DOI:** 10.3390/plants14060846

**Published:** 2025-03-08

**Authors:** Gabriela Valentina Ciobotaru, Iacob-Daniel Goje, Cristina Adriana Dehelean, Corina Danciu, Ioana Zinuca Magyari-Pavel, Elena-Alina Moacă, Delia Muntean, Ilinca Merima Imbrea, Veronica Sărățeanu, Georgeta Pop

**Affiliations:** 1 Medical Semiology Clinic, Department V. Internal Medicine 2, Faculty of Medicine, “Victor Babes” University of Medicine and Pharmacy, 2 Eftimie Murgu Sq., 300041 Timisoara, Romania; vaciobotaru@gmail.com (G.V.C.); muntean.delia@umft.ro (D.M.); 2 Municipal Clinical Emergency Hospital, 5 Gh. Dima, 300079 Timisoara, Romania; 3 Department of Toxicology, Drug Industry, Management and Legislation, Faculty of Pharmacy, “Victor Babes” University of Medicine and Pharmacy, 2 Eftimie Murgu Sq., 300041 Timisoara, Romania; cadehelean@umft.ro (C.A.D.); alina.moaca@umft.ro (E.-A.M.); 4 Research Centre for Pharmaco-Toxicological Evaluation, “Victor Babes” University of Medicine and Pharmacy, 2nd Eftimie Murgu Square, 300041 Timisoara, Romania; 5 Department of Pharmacognosy-Phytotherapy, Faculty of Pharmacy, “Victor Babes” University of Medicine and Pharmacy, Eftimie Murgu Square, No. 2, 300041 Timisoara, Romania; corina.danciu@umft.ro (C.D.); ioanaz.pavel@umft.ro (I.Z.M.-P.); 6 Research and Processing Center for Medicinal and Aromatic Plants, “Victor Babes” University of Medicine and Pharmacy, Eftimie Murgu Square, No. 2, 300041 Timisoara, Romania; 7 Faculty of Engineering and Applied Technologies, University of Life Sciences “King Mihai I” from Timisoara, Calea Aradului 119, 300645 Timisoara, Romania; ilinca_imbrea@usvt.ro; 8 Department of Crop Science, Faculty of Agriculture, University of Life Sciences “King Mihai I” from Timisoara, Calea Aradului 119, 300645 Timisoara, Romania; veronica_sarateanu@usvt.ro

**Keywords:** essential oil, *Lamiaceae*, antioxidant activity, antimicrobial activity, cytotoxic activity, anti-migratory activity

## Abstract

This study aims to highlight the therapeutic potential of some *Lamiacea* essential oils (EOs). For this purpose, eight EOs, including two from *Lavandula angustifolia* Mill. cultivated in Romania and Spain (LA1 and LA2), *Salvia officinalis* L. (SO), *Lavandula hybrida* Balb. ex Ging (LH), *Salvia sclarea* L. (SS), *Mentha smithiana* L. (MS), *Perovskia atriplicifolia* Benth. (PA), and *Mentha x piperita* L. (MP), were evaluated in vitro in terms of antioxidant, cytotoxic, antimicrobial, and anti-migratory activities. As regards the antioxidant capacity, expressed as the EO concentration that produces 50% of the maximum effect (IC_50_ value), the EOs obtained from the cultivated plants of the *Lamiaceae* family are ordered as follows: LA2 ˃ LA1 ˃ LH > MP > MS > SO > SS > PA. For the determination of antimicrobial activity, the reference strains used for testing were *Salmonella enterica* serotype *typhimurium*, *Shigella flexneri* serotype 2b, *Enterococcus faecalis*, *Escherichia coli*, *Klebsiella pneumoniae*, *Pseudomonas aeruginosa*, *Staphylococcus aureus*, *Candida albicans*, and *Candida parapsilosis*. The most intense inhibitory effect was observed in EOs of MS and MP on all tested microbial strains. The cytotoxic and anti-migratory activity of EOs was tested on two melanoma cell lines (A375 and B164A5) and on a healthy keratinocyte line (HaCaT). EOs LA1 and MP manifested the highest selectivity on the analysed tumoural cells, by reducing their migration in comparison with the control, proving to have therapeutic potential.

## 1. Introduction

According to the World Health Organization [1], more than 80% of the population prefers traditional medicine as the basis of any treatment. This alternative to conventional drugs might offer a lot of opportunities for researchers to study the plants that can be found in the vicinity of the cities where they work and take advantage of their medicinal properties. It has been observed that the essential oils resulting from their studies have beneficial properties with therapeutic uses [2,3,4].

Essential oils (EOs) are produced by plants as protection against pests and insects [5]. Many species from the *Lamiaceae* family are known to contain EOs used in the medical, cosmetic, and food industries [6]. EOs contain a mixture of active compounds, the most numerous of which are terpenoids and their oxygenated derivatives [7,8,9]. The chemical composition of EOs can be influenced by the geographical location of the plant species, the cultivation technology, and the conditioning and extraction method used [5]. They may also produce adverse or toxic effects on humans, as no rigorous tests are currently established to determine the safety of EO administration [5].

The pharmacological properties of EOs derived from *Lamiaceae* species have garnered significant attention due to their diverse biological activities, including antimicrobial, antioxidant, anti-inflammatory, and anticancer effects [6,10,11]. The genotoxicity of EOs has been evaluated several times over the years [12,13,14,15,16]. EOs from *Lamiaceae* species are rich in bioactive compounds, predominantly monoterpenes and sesquiterpenes, which contribute to their therapeutic properties [7,8,9]. These EOs are increasingly recognized for their potential applications in pharmaceuticals, food preservation, and cosmetics due to their ability to scavenge free radicals and mitigate oxidative stress. The antioxidant properties of *Lamiaceae* EOs are noteworthy. Research indicates that these oils can effectively scavenge free radicals, thereby mitigating oxidative stress, which is implicated in various chronic diseases [17,18].

The antioxidant activity of EOs is often attributed to their chemical composition, particularly the presence of phenolic compounds, known for their free-radical-scavenging abilities [7]. In addition, EOs from various species have demonstrated synergistic antioxidant effects when combined, indicating that the interactions between different EOs can enhance their overall efficacy [19]. The EOs from various *Lamiaceae* species exhibit varying degrees of antioxidant activity, suggesting that the specific phytochemical profiles of these oils play a crucial role in their antioxidant effectiveness [20]. The total phenolic content of EOs has been correlated with their antioxidant activity, with higher total phenolic content values often leading to enhanced free-radical-scavenging capabilities [21,22]. For example, the EOs of *Lavandula angustifolia* have been shown to exhibit strong antioxidant activity, which is attributed to its high content of phenolic compounds [9]. Research also indicates that EOs can serve as natural alternatives to synthetic antioxidants in food products, effectively prolonging shelf life and maintaining quality by preventing oxidative deterioration [23,24].

The antimicrobial and antimutagenic activities of EOs belonging to the *Lamiaceae* family underlie many applications, including pharmaceutical, medicinal, cosmetic, and even processed foods applications, due to the bioactive compounds contained in them [12,25]. Studies have shown that EOs from various species of the *Lamiaceae* family possess potent antifungal properties, particularly against *Candida albicans* and *Staphylococcus aureus* [26,27]. In addition, *Lamiaceae* EOs have demonstrated anti-inflammatory effects, which can be beneficial in treating inflammatory conditions [8,28]. These oils have been explored for their potential in wound healing and tissue regeneration, further underscoring their therapeutic versatility [28,29].

The application of *Lamiaceae* EOs extends beyond traditional medicine into modern pharmaceutical and agricultural industries. Their insecticidal properties have been documented, showing efficacy against agricultural pests [29,30]. This suggests that these oils could serve as natural alternatives to synthetic pesticides, promoting sustainable agricultural practices.

In the context of the above, the present research aims at investigating potential therapeutic uses of some *Lamiaceae* EOs. For this purpose, tests regarding the antioxidant, antimicrobial, cytotoxic, and anti-migratory activity on carcinogenic cells were performed using eight EOs from cultivated plants belonging to the *Lamiaceae* family, including two from *Lavandula angustifolia* Mill. (LA1 cultivated in Romania and LA2 cultivated in Spain), *Salvia officinalis* L. (SO), *Lavandula hybrida* Balb. ex Ging (LH), *Salvia sclarea* L. (SS), *Mentha smithiana* L. (MS), *Perovskia atriplicifolia* Benth. (PA), and *Mentha x piperita* L. (MP), respectively.

The results obtained proved the potential therapeutic capacity of the analysed EOs from antioxidant, antimicrobial, and antitumoural perspectives, confirming the previous results in the literature [5,12,14,16,17,18,20]. This fact opens the opportunity for more complex and detailed investigations.

## 2. Results

### 2.1. DPPH Radical-Scavenging Assay of EOs Obtained from Cultivated Medicinal Species Belonging to the Lamiaceae Family

The antioxidant activity of the eight samples of EOs obtained from cultivated species of the *Lamiaceae* family are expressed using IC_50_ values, which are detailed in Table 1. The IC_50_ value represents the half-maximal inhibitory concentration of each EO, where 50% of its maximal effect is observed, which refers to the potency required to obtain a 50% antioxidant effect.

The antioxidant capacity percentage obtained for all the EO samples represents an average of three measurements ± standard deviation (SD). Further, by linear regression analysis, the IC_50_ value was calculated. It can be observed that all the analysed samples show an antioxidant capacity from moderate to high, in some cases (*Lavandula angustifolia* Mill. and *Lavandula hybrida* Balb. ex Ging). Therefore, regarding the concentration tested (200 μg/mL), the antioxidant potential of each essential oil is ordered as follows: LA2 ˃ LA1 ˃ LH > MP > MS > SO > SS > PA.

### 2.2. Antimicrobial Action of the Analysed EOs

#### 2.2.1. Analysis of the Diameters of Inhibition Zones Obtained by the Disc Diffusion Method

Table 2 shows the zones of inhibition for the analysed EOs. The zones were obtained by the disc-diffusion method. For all bacterial strains, a Gentamicin micro-tablet of 10 µg, except 120 µg for the *Enterococcus faecalis* strain, was used as a control. For fungal strains of *Candida*, a 10 µg Fluconazole micro-tablet was used as a control.

EOs with an inhibition zone diameter greater than 15 mm were considered to have antimicrobial activity and were further tested by the dilution method.

For the SO oil, no antimicrobial activity was recorded, while LA1, PA, LH, LH, LA2, and SS oils were active only on Gram-positive cocci and fungi. The other EOs (MS and MP) had inhibitory activity on all reference strains tested.

Table 3 presents the matrix of the Euclidean distances calculated for the measured diameters of the paired samples of EOs used for the determination of similarities in antimicrobial activity.

**Figure 1:**
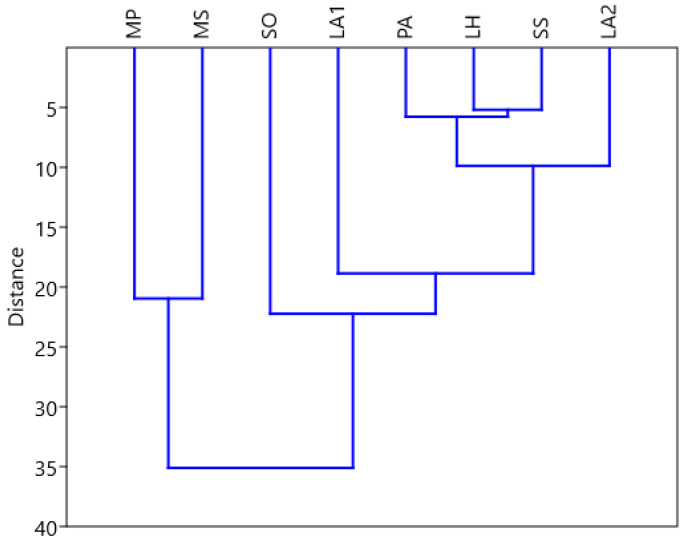
Hierarchical cluster for describing similarity in the activity of the studied plant samples (Source: authors’ own graphical representation of experimental data using PAST 4.03).

The dendrogram constructed using Euclidean distances with paired groups has a cophenetic correlation coefficient with a value of 0.91, indicating that, to a large extent, the dendrogram keeps the distances in agreement with the empirical data. The EOs LH and SS have close activity in size. Also, even PA and LA2 have similar activities. Important differences are observed between MS and MP, each of which is relative to SO. The groups formed are represented in Figure 1.

**Figure 2:**
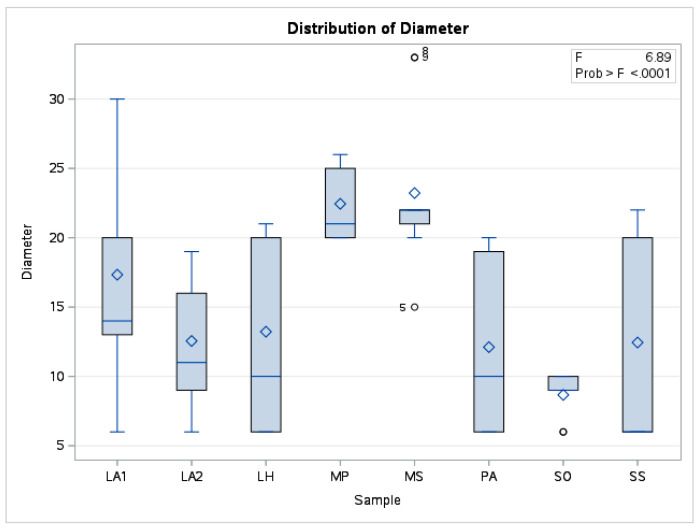
Boxplot distribution of measured diameter values for the analysed EO samples (Source: authors’ own graphical representation of experimental data using SAS Studio).

The mean values corresponding to the EOs studied then formed the basis for prioritising the sample types. Together with other statistical indicators, the data are presented in the boxplot in Figure 2.

#### 2.2.2. Determination of Minimum Inhibitory Concentration and Minimum Concentration

The EO/DMSO dilutions were as follows: 40, 20, 10, 5, and 2.5 mg/mL. The values for the MIC (minimum inhibitory concentration) and MBC (minimum bactericidal concentration)/MFC (minimum fungicidal concentration) are given in Table 4.

The EO extracted from LA1 exhibits antimicrobial activity against Gram + cocci (*S. aureus* 20 mm and *E. faecalis* 19 mm) and fungi (*C. albicans* 30 mm and *C. parapsilosis* 30 mm). The main chemical compounds of the LA EO are linalool (22.11%) and linalyl acetate (20.384%) in approximately equal proportions.

LH and SS exhibited congruent antimicrobial activity with an inhibition diameter of about 20–22 mm for Gram + cocci (*S. aureus* and *E. faecalis*) and fungi (*C. albicans* and *C. parapsilosis*).

The main chemical compounds of the LH EO are linalool (35.86%) and eucalyptol (17.84%). As for the SS strain, linalyl acetate and linalool are found in 80%, which supports the antimicrobial activity of these compounds.

The EO extracted from LA2 is found to have weaker antimicrobial activity on Gram + cocci (*S. aureus* 16 mm and *E. faecalis* 15 mm) and fungi (*C. albicans* 19 mm and *C. parapsilosis* 18 mm) compared to the EOs extracted from LH and SS.

The MS EO exhibited antibacterial and antifungal activity on all strains tested, with a maximum zone of inhibition on fungi (33 mm *C. albicans* and *C. parapsilosis*).

The EO extracted from the strain of SO had a diameter of the zone of inhibition of less than 15 mm. Thus, it was considered to have no antimicrobial activity and was not further tested by the dilution method.

The major compound of the EO extracted from SO is beta-thujone (16.84%), which does not seem to induce antibacterial and antifungal activity on the tested species (*K. pneumoniae*, *S. flexneri*, *S. enterica*, *E. coli*, *P. aeruginosa*, *S. aureus*, *E. faecalis*, *C. albicans*, *C. parapsilosis*).

### 2.3. Antitumoural Effects of the EOs

#### 2.3.1. Analysis of the Cytotoxic Effect of the EOs

In this part of the study, the effect of the EOs was analysed on two melanoma cell lines and on a keratinocyte line at 24 h post-stimulation. Two concentrations (50 and 150 µg/mL) of each sample were tested. All calculations were based on the solvent used to prepare the solutions—dimethylsulfoxide (DMSO). The highest DMSO concentration tested was 0.15%.

**Figure 3:**
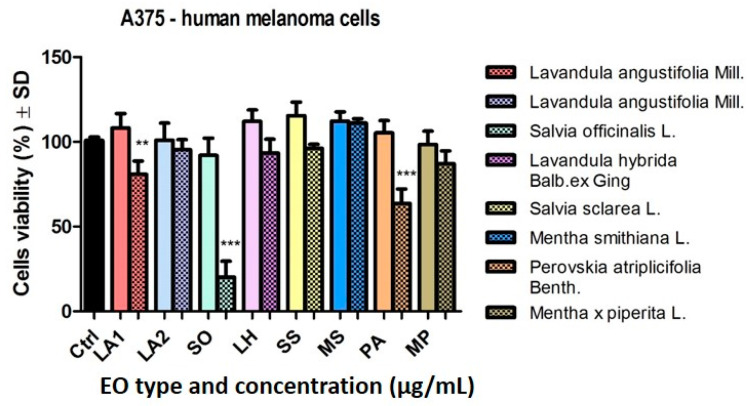
A375 human melanoma cell viability, after stimulation with the EOs (50 and 150 μg/mL—clear vs. dotted texture of the colour fill) for 24 h. Data are expressed as mean ± SD (*** *p* < 0.001; ** *p* < 0.01). The comparison between groups was performed using the One-way ANOVA test followed by Dunnett’s post-test.

Figure 3 shows the viability of A375 human melanoma cells after stimulation with the EOs. At the lowest dose tested, 50 µg/mL, the samples had no significant effect on cell viability. The graph shows a slight reduction in tumour cell viability when stimulated with the EOs obtained from LA2 and SO.

Increasing the dose used (150 µg/mL) led to a decrease in the viability of A375 tumour cells following stimulation with certain samples (Figure 3). The most significant result was observed in the case of the SO EO sample (*** *p* < 0.001). The viability of tumour cells also decreased in the PA EO sample (*** *p* < 0.001) and to a lesser extent in the LA1 EO sample (** *p* < 0.01). By increasing the dose, a greater decrease in cell viability was observed for the LA1 sample compared to LA2, but without statistical significance, which is different from the results obtained for the same samples tested at a low dose.

In the case of the MP EO sample, a decrease in cell viability (87.1 ± 7.5% versus the solvent DMSO 0.15%) was observed. The other three EO samples (LH, SS, and MS) at this dose had no effect on human melanoma cells.

**Figure 4:**
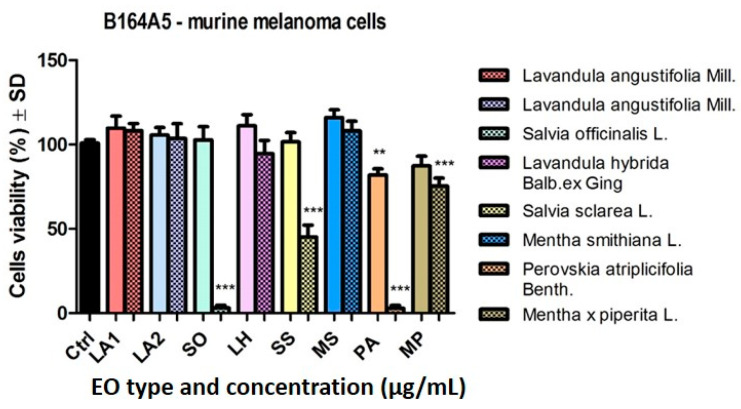
B164A5 murine melanoma cell viability, after stimulation with the EOs (50 and 150 μg/mL—clear vs. dotted texture of the colour fill) for 24 h. Data are expressed as mean ± SD (*** *p* < 0.001; ** *p* < 0.01). The comparison between groups was performed using the One-way ANOVA test followed by Dunnett’s post-test.

The effect of the aforementioned samples was also evaluated on the murine melanoma cell line B164A5 (Figure 4). At the low dose tested (50 µg/mL), the EO sample from PA reduced tumour cell viability (81.91 ± 3.6% vs. DMSO). The MP EO also showed a slight reduction in cell viability. The other samples had no major influence at 50 µg/mL on this cell line.

By increasing the dose used (150 μg/mL) (Figure 4), there was a significant reduction in cell viability after stimulation with the EO samples of SO and PA (*** *p* < 0.001). On B164A4 cells, it was observed that stimulation with EO samples of SS and MP led to a decrease in murine melanoma cell line viability, whereas at the same dose, these samples had no significant effect on the human melanoma cell line.

**Figure 5:**
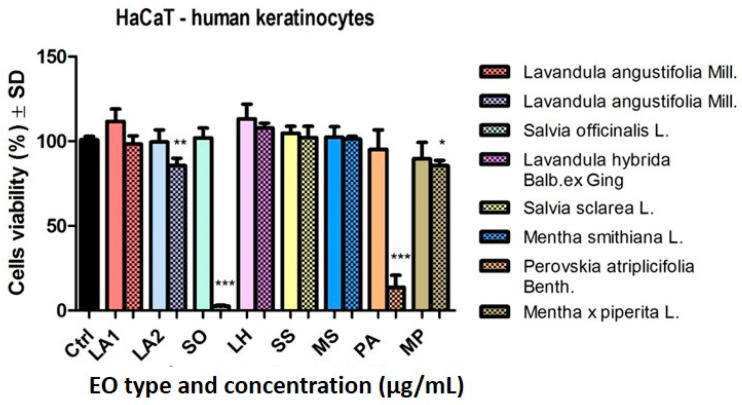
HaCaT human keratinocyte viability after stimulation with the EOs (50 and 150 μg/mL—clear vs. dotted texture of the colour fill) for 24 h. Data are expressed as mean ± SD (*** *p* < 0.001; ** *p* < 0.01 and * *p* < 0.050). The comparison between groups was performed using the One-way ANOVA test followed by Dunnett’s post-test.

The effect of EOs was also tested on a non-tumour line, namely, HaCaT keratinocytes. At 50 μg/mL, the samples did not affect significantly cell viability (Figure 5).

At the highest dose tested, the EOs of SO and PA produced a significant decrease (*** *p* < 0.001) in cell viability. The EOs of LA2 (** *p* < 0.01) and MP (* *p* < 0.05) also elicited a reduction in keratinocyte viability (Figure 5).

#### 2.3.2. Analysis of the Anti-Migratory Effect of the EOs Using the Scratch Technique

Tumour cells exhibit an increased ability to migrate and this property has an important role in tumour progression.

In this experiment, the anti-migratory effect of EOs was determined on two melanoma cell lines and on a keratinocyte line. The lowest dose (50 µg/mL) of each sample was tested, as it is desired to assess whether a reduction in the migratory ability of tumour cells occurs.

##### Determination of the Anti-Migratory Effect of the EOs on A375 Human Melanoma Cell Line

**Figure 6:**
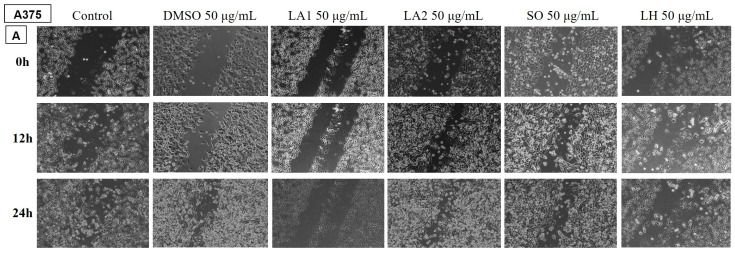
The anti-migratory effect of the tested EOs (50 μg/mL) (A—LA1; LA2; SO and LH) on human melanoma cell line A375. The images were taken at 0, 12, and 24 h after stimulation.

**Figure 7:**
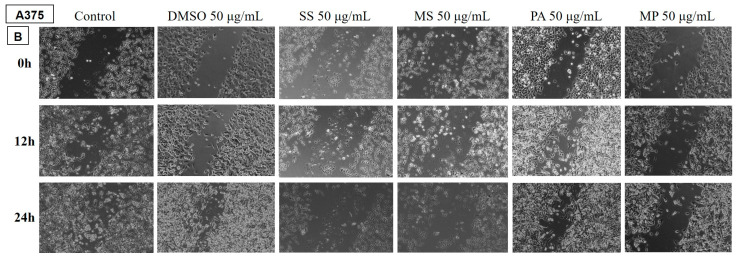
The anti-migratory effect of the tested EOs (50 μg/mL) (B—SS; MS; PA and MP) on human melanoma cell line A375. The images were taken at 0, 12, and 24 h after stimulation.

In Figure 6 and Figure 7, the effect of the EOs belonging to the *Lamiaceae* family is shown. The images were compared with a control (untreated cells grown in culture medium) and DMSO (cells stimulated with the solvent used to obtain stock solutions) at the same concentration.

The tested samples reduced the migration of A375 tumour cells compared to the control and DMSO groups, but to a lesser extent for the EOs of LA2, SO, and PA.

Figure 6 shows the effect of LA1 on A375 melanoma cells. It can be seen that at the dose of 50 µg/mL, the sample reduces the migratory ability of tumour cells compared to the control and DMSO.

On the other hand, the 50 µg/mL EO samples of LA2, SO, and PA do not inhibit the migratory capacity of the human melanoma cell line, highlighting that at this dose, they have no antitumour effect.

All other tested samples, including LH, SS, MS, and MP, produced a reduction in tumour cell migration compared to the control and DMSO groups, proving to have beneficial properties against melanoma.

##### Determination of the Anti-Migratory Effect of the EOs on B164A5 Murine Melanoma Cell Line

**Figure 8:**
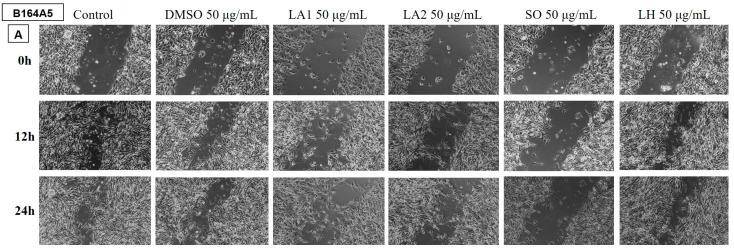
The anti-migratory effect of the tested EOs (50 μg/mL) (A—LA1; LA2; SO and LH) on murine melanoma cell line B164A4. The images were taken at 0, 12, and 24 h after stimulation.

**Figure 9:**
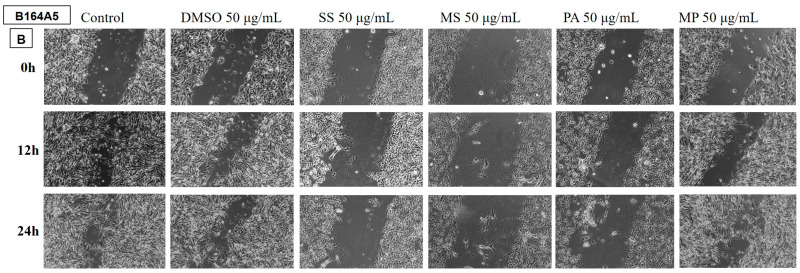
The anti-migratory effect of the tested EOs (50 μg/mL) (B—SS; MS; PA and MP) on murine melanoma cell line B164A4. The images were taken at 0, 12, and 24 h after stimulation.

The effect of the EOs obtained from different species belonging to the *Lamiaceae* family was also tested on the B164A5 murine melanoma cell line (Figure 8 and Figure 9). The images were compared with the control and DMSO at the same concentration. Again, the lower dose of 50 µg/mL was tested for all samples.

The LH and MP EO samples did not have an anti-migratory effect on the tumour cells, the migration rate for these samples being similar to the control.

The rest of the tested samples significantly reduced the migration ability of murine melanoma cells.

##### Determination of the Anti-Migratory Effect of the EOs on HaCaT, Human Keratinocyte

**Figure 10:**
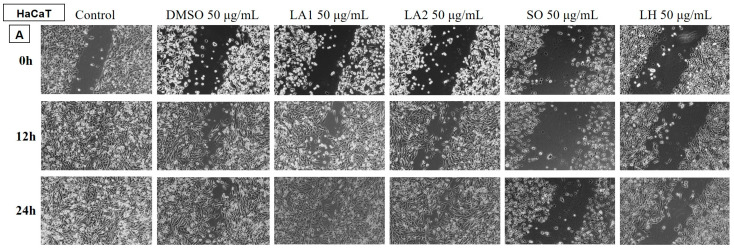
The anti-migratory effect of the tested EOs (50 μg/mL) (A—LA1; LA2; SO and LH) on the HaCaT cell line, keratinocytes. The images were taken at 0, 12 and 24 h after stimulation.

**Figure 11:**
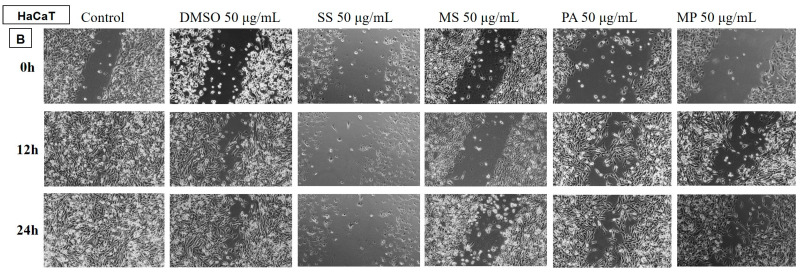
The anti-migratory effect of the tested EOs (50 μg/mL) (B—SS; MS; PA and MP) on the HaCaT keratinocyte cell line. The images were taken at 0, 12, and 24 h after stimulation.

The EOs (50 µg/mL) were also tested on a non-tumour cell line, HaCaT human keratinocyte (Figure 10 and Figure 11), to assess whether it showed selectivity, i.e., whether it affected only tumour cells and did not affect the migratory capacity of healthy cells. The images were compared with the control and DMSO at the same concentration.

The obtained data showed that the EO samples of LA1, LA2, PA, and MP stimulated the migration of human keratinocytes, similar to the control group, while the rest of the tested EO samples reduced the migration capacity of HaCaT cells.

In comparison with the data obtained on the human melanoma cell line, it can be found that the EOs of LA1 and MP reduced the migration capacity of tumour cells and, at the same dose, stimulated the migration of human keratinocytes, proving to be selective samples with beneficial effects against human melanoma.

## 3. Discussion

### 3.1. Antioxidant Capacity of EO

The antioxidant capacity refers to the ability of a substance to neutralize free radicals and mitigate oxidative stress, which is linked to various chronic diseases and ageing processes. Oxidative stress is considered to be an imbalance in redox homeostasis between the excessive production of reactive oxygen species (ROS) and a deterioration in antioxidant capacity. This can occur in various diseases such as cancer, diabetes, vascular and brain dysfunction, ageing, etc. ROS are a diverse group of highly reactive molecules that play critical roles in various biological processes. Their reactivity is primarily attributed to the presence of unpaired electrons in their molecular structure, which makes them capable of engaging in chemical reactions that can lead to oxidative stress and cellular damage. ROS can be classified into two main categories: free radicals, which possess unpaired electrons, and non-radical species, such as hydrogen peroxide (H_2_O_2_) [31]. The most common free radicals include superoxide (O_2_^−^) and hydroxyl radicals (·OH), both of which are known for their high reactivity and ability to oxidize a wide range of biological macromolecules, including lipids, proteins, and DNA [32]. The generation of ROS occurs as a natural by-product of aerobic metabolism, particularly during mitochondrial respiration, where electrons are transferred through the electron transport chain [33,34]. Under normal physiological conditions, ROS are produced in controlled amounts and are kept in balance by antioxidant systems, which include enzymes like superoxide dismutase and catalase, as well as small molecules like glutathione [32]. However, when the production of ROS exceeds the capacity of these antioxidant defences, it leads to oxidative stress [35,36]. The reactivity of ROS is not solely detrimental; they also serve important signalling functions within cells. Low concentrations of ROS can act as second messengers in various signalling pathways, influencing processes such as cell growth, differentiation, and immune responses [37]. This dual role of ROS—acting as both signalling molecules and potential agents of damage—highlights their complexity in biological systems. The balance between their beneficial and harmful effects is crucial for maintaining cellular homeostasis and responding to environmental stresses [38].

The antioxidant capacity is typically quantified using several standardized assays that measure the effectiveness of antioxidants in different contexts, including biological systems and food products. The properties of EOs have been most widely evaluated using the 2,2-diphenyl-1-picrylhydrazyl (DPPH) method, which measures the ability of these oils to donate hydrogen and neutralize free radicals [39,40]. In the present research, we analysed the antioxidant capacity of eight EOs obtained from cultivated *Lamiaceae* species by the classical DPPH method. Our results showed a moderately high antioxidant capacity, expressed in terms of IC_50_ values, for the EOs analysed. For instance, the EOs from *Lavandula angustifolia* Mill (LA1 and LA2) exhibit the highest IC_50_ value (15.84 ± 0.92 μg/mL and 13.28 ± 0.67 μg/mL). Our outcomes are comparable with those reported in the literature [41,42,43,44], highlighting the significant biological activities of these EOs, particularly in antioxidant and antimicrobial capacities. The differences between our results and those reported in the literature [41,42,43,44] came from the variability in the chemical composition of *Lavandula angustifolia* EOs, which has also been documented [45,46].

The EO of *Lavandula hybrida* is characterized by a rich profile of terpenes, predominantly linalool and linalyl acetate, which are known for their ability to neutralize free radicals, thereby enhancing the oil’s overall antioxidant capacity [47]. The presence of phenolic compounds in EOs has also been linked to antioxidant activity, further supporting the potential of *Lavandula hybrida* in this regard [48]. In comparative studies, the antioxidant capacity of *Lavandula hybrida* has been evaluated alongside other species [42,43,49,50]. Our findings revealed an IC_50_ value of 19.39 ± 0.98 μg/mL, which indicates, as in other research studies, the ability of the *Lavandula hybrida* EO to scavenge free radicals, although specific IC_50_ values were not detailed in the findings in [51].

The EO from *Salvia officinalis* has shown varying IC_50_ values across different studies. It has been stated that the antioxidant properties of the *S. officinalis* EO are often attributed to the presence of phenolic compounds and terpenes, which are prevalent in the oil’s composition [52,53]. Our findings revealed an IC_50_ value of 94.73 ± 1.18 μg/mL for the EO of *Salvia officinalis* L. and 109.28 ± 1.34 μg/mL for the EO of *Salvia sclarea* L. In the case of *Salvia officinalis* EOs, the antioxidant properties are often attributed to the presence of phenolic compounds and terpenes, which are prevalent in the oil’s composition [52,53,54]. The variability regarding the results is due to the *Salvia officinalis* EOs’ chemical composition, which depends on factors such as geographical location and extraction methods; these factors can influence the observed IC_50_ values and overall efficacy of the essential oil [55,56]. The antioxidant capacity of the *Salvia sclarea* L. essential oil has been investigated in various studies, revealing significant insights into its bioactive properties [57,58,59,60]. In the study reported by Aćimović et al., the essential oil of *S. sclarea* exhibited an IC_50_ value of approximately 400 µg AAE/mL in the DPPH assay, indicating its capacity to neutralize free radicals [61]. This value, while higher than that obtained in the present study (109.28 ± 1.34 μg/mL), still demonstrates a notable antioxidant potential of *S. sclarea* EO.

While specific IC_50_ values for *Mentha smithiana* essential oil were not directly reported in the literature, related studies on other *Mentha* species provide a comparative context, indicating that its IC_50_ values can vary widely depending on the methodology used [62,63,64,65]. The results of the present study regarding the *Mentha* species’ EO agree with the literature and are even better [66,67,68,69]. We obtained an IC_50_ value of 45.75 ± 1.06 μg/mL for the EO obtained from the *Mentha piperita* cultivated medicinal plant and an IC_50_ value of 65.78 ± 1.21 μg/mL for the EO from *Mentha smithiana* cultivated medicinal plant.

The EOs from *Perovskia atriplicifolia* Benth. have been shown to contain a diverse array of phytochemicals, primarily monoterpenes and sesquiterpenes, which contribute to their bioactivity [70,71]. Although specific IC_50_ values for *Perovskia atriplicifolia* EOs were not detailed in the available literature, related studies on other species within the *Lamiaceae* family suggest that EOs from this group typically exhibit strong antioxidant properties [71,72]. Our findings revealed an IC_50_ value of 186.84 ± 1.56 μg/mL for the EO of the *Perovskia atriplicifolia* cultivated medicinal plant, a value which is comparable with the literature [71,72]. Overall, the antioxidant activity of EOs is often linked to their chemical constituents. The variability in antioxidant capacity is influenced by the chemical composition of the EOs, which is affected in turn by environmental factors.

### 3.2. Antimicrobial Effect of EO

Our results proved that the LA1 EO had antibacterial properties against Gram + cocci *S. aureus* and *E. faecalis* and antifungal activity against *C. albicans* and *C. parapsilosis*.

In the literature, it is mentioned that the LA EO is effective in the inhibition and control of bacterial strains and could be used as a natural antibacterial agent [73]. Our results showed a low antimicrobial activity on Gram + cocci and fungi compared to *Lavandula hybrida*. The results obtained are in agreement with the literature data, as published by de Rapper et al. [74]; the complete chemical profile of the EO extracted from *Lavandula angustifolia* Mill coincides with our results. Moreover, it was demonstrated that the lavender EO exhibits significant antibacterial activity against various pathogens, which is crucial for its application in food safety and medicinal products [75]. The EO’s effectiveness in inhibiting bacterial growth is attributed to its major components, including linalool and linalyl acetate, which possess inherent antimicrobial properties [76,77]. In addition, studies have shown that *Lavandula hybrida* exhibits varying inhibitory concentration levels against different microorganisms [51,78,79].

LH and SS EOs manifested antibacterial activity for Gram + cocci (*S. aureus* and *E. faecalis*) and antifungal activity (*C. albicans* and *C. parapsilosis*), due to their chemical composition [53,59]. The antimicrobial efficacy of the *Salvia officinalis* essential oil has also been extensively studied [80,81]. As regards the EO of *S. sclarea*, studies have reported MIC values against *Staphylococcus aureus* and *Staphylococcus epidermidis* as low as 5.0 mg/mL [82,83]. Additionally, the essential oil has shown effectiveness against multidrug-resistant strains, further emphasizing its potential as a natural antimicrobial agent [82,84].

The antimicrobial properties of *Mentha* EOs have been extensively documented. The essential oil from *Mentha piperita* exhibited significant antimicrobial activity, with MIC values reported as low as 3.75 µL/mL against various pathogens [85]. In our research, the MS EO demonstrated antimicrobial properties on all pathogenic strains tested, both bacterial and fungal, according to other reported studies [86,87,88,89,90,91]. The synergistic effects of the *Mentha piperita* essential oil with conventional antifungal agents have also been explored, suggesting that combining these oils with existing treatments could enhance their efficacy and reduce the likelihood of resistance [92]. Therefore, all the findings reported underscore the potential of *Mentha piperita* EOs as effective antimicrobial agents, with specific IC_50_ values that vary based on the concentration and target microorganism. The ongoing investigation into the chemical composition and environmental influences of these oils will further elucidate their practical applications in medicine and agriculture.

The results obtained in our study show that the SO EO has no antimicrobial activity on the tested bacterial and fungal strains. In other research, the antimicrobial activity of the endemic population of *Salvia officinalis* from Egypt, with a major composition of camphor (25.1%), a-thujone (22.2%), and b-thujone (17.7%), was tested by chemical analysis. It was found to have antimicrobial activity against *K. pneumoniae*, *S. aureus*, *E. coli,* and *C. albicans*, while no effect was found against *P. aeruginosa* [93].

As regards the *Perovskia atriplicifolia* medicinal plant investigated, the EOs have demonstrated promising antimicrobial activities against various pathogens. The presence of active compounds in the EOs suggests potential applications in both food preservation and therapeutic contexts. EOs from related species have been noted for their effectiveness against *Escherichia coli* and *Staphylococcus aureus*, which are common bacterial pathogens [94,95]. The IC_50_ values for these activities are crucial for determining the efficacy of these oils in clinical and agricultural applications, although specific values for *Perovskia atriplicifolia* remain to be fully elucidated.

### 3.3. Cytotoxic and Anti-Migratory Effects of the EOs

The results obtained indicate that the EO samples with the highest cytotoxic activity are *Salvia officinalis* L. (SO) and *Perovskia atripicifolia* Benth. (PA). The EOs analysed show a dose-dependent cytotoxic effect; the most significant data are obtained after 24 h cell stimulation, with a dose of 150 μg/mL. These samples do not show selectivity against tumour cells because, at high doses, they also reduced keratinocyte viability.

In terms of anti-migratory capacity, the obtained data indicate that two EO samples, LA1 and MP, reduced the migration capacity of melanoma to a higher extent in comparison with the control. A comparative evaluation of the results obtained on the human melanoma cell line and human keratinocytes showed that the LA1 and MP samples showed increased selectivity; they stimulated the migration of healthy cells and inhibited the migration capacity of tumour cells, respectively.

Different types of extracts and EOs obtained from plants belonging to the *Lamiaceae* family are frequently studied for their antitumour effect, showing positive effects against melanoma [96,97]. Cocan et al. [97] evaluated the biological activity of an ethanolic extractive solution obtained from *Salvia officinalis* L. The authors determined the effect of the extractive solution (at concentrations of 50 and 100 µg/mL) on A375 human melanoma cell lines and the B164A5 murine melanoma cell line. All samples produced a significant dose-dependent decrease in tumour viability.

In another experiment, the EOs obtained from *Salvia officinalis* L. and *Thymus vulgaris* L. species were tested for antifungal and antiproliferative activity on two melanoma cell lines, A375 and B164A5 [98]. The EO obtained from *Salvia officinalis* L. produced a significant inhibition of tumour proliferation; at a dose of 100 µg/mL, it inhibited proliferation by 50.5% for the murine melanoma line and by 47.5% for the human melanoma line. The *Thymus vulgaris* L. EO inhibited melanoma cell proliferation but to a lesser extent. Regarding the chemical composition, the EO from *Salvia officinalis* L. had caryophyllene, camphene, eucalyptol, and β-pinen as its main compounds [98].

Our results in terms of viability are in agreement with those obtained by Alexa et al. [98]; the EO obtained from *Salvia officinalis* L. (SO) produced a significant decrease in cell viability at the high dose tested (150 µg/mL) in both tumour cell lines. On the other hand, the chemical composition of the EO was different in our case, with the EO from SO having D-limonene, beta-thujone, and alpha-thujone as its main compounds.

Our findings show that the EOs with high antioxidant capacity had better antimicrobial effects; this relationship should be further investigated, as it is useful in the selection of EOs with therapeutic potential. The results obtained on the antimicrobial effect of the EOs obtained from *Lamiaceae* species can represent therapeutic alternatives with natural bioactive compounds that may be effective in the treatment of some bacterial and fungal infections resistant to synthetic substances.

Some of the analysed EOs have inhibited the proliferation and migration of melanoma cell lines, and similar results are present in the literature. This path can be approached in future research for the identification of the bioactive compounds from the EOs and other factors that can influence the therapeutic potential.

## 4. Materials and Methods

The analysed EOs were extracted from medicinal plants cultivated in two geographical locations, the Young Naturalists Station Timisoara and the town of Rivas in Spain during the period 2013–2016. The plants come from organic crops and were harvested during the flowering period at midday. The species used for the essential oil extraction belongs to the *Lamiaceae* family, including *Lavandula angustifolia* Mill. (LA1—cultivated at Timișoara), *Lavandula angustifolia* Mill. (LA2—cultivated in Rivas), *Salvia officinalis* L. (SO), *Lavandula hybrida* Balb. ex Ging (LH), *Salvia sclarea* L. (SS), *Mentha smithiana* L. (MS), *Perovskia atriplicifolia* Benth. (PA), and *Mentha x piperita* L. (MP).

The collected plants were identified and stored in the Department of Medicinal Plants, University of Life Sciences “King Mihai I” from Timisoara, except the LA2 sample, which was processed and analysed in Spain. Each plant received a voucher number.

### 4.1. Materials Used

#### 4.1.1. EO Extraction and Composition Analysis

For the medicinal plant species studied, the amount of essential oil obtained from 100 g of the dried herb was determined and the drying yield was calculated. The extraction of the EOs was performed with a Clevenger Apparatus by steam distillation (Table 5). The amount of volatile oil obtained from each species was determined, and the drying yield was also calculated, as shown in Table 5.

The composition of EOs was determined by the chromatographic method and by a GC-MS analysis of the samples at the Laboratory of “Aurel Vlaicu” University of Arad, Romania; the results are presented in Table A1, Table A2, Table A3, Table A4, Table A5, Table A6, Table A7 and Table A8 from Appendix A. The EO constituents were determined based on their mass spectra using the NIST 14 library [99] and the Wiley 09 library [100].

#### 4.1.2. Reagents

The determination of antioxidant activity was carried out with ethyl alcohol 96% (*v*/*v*), which was purchased from Chemical Company SA, Iasi, Romania, ascorbic acid, which was purchased from Lach-Ner (Neratovice, Czech Republic), and DPPH (TBF5255V), which was purchased from Sigma Aldrich (St. Louis, MI, USA). For the determination of microbial activity, DMSO was used as a solvent for the samples purchased from Sigma Aldrich. Gentamicin and Fluconazole micro-tablets were obtained from Bio-Rad, Marnes-la-Coquette (France). Agar Columbia +5% sheep blood and Sabouraud with chloramphenicol, respectively, were purchased from bioMerieux (Marcy-l’Étoile, France).

#### 4.1.3. Microbial Strains

The reference strains used (Table 6) for testing were selected to represent microbial species that can colonise the intestinal tract.

### 4.2. Methods Used

#### 4.2.1. DPPH Radical-Scavenging Assay

The 2,2-diphenyl-1-picrylhydrazyl (DPPH) method is commonly used to determine the antioxidant activity of different types of extracts/samples [101]. The experimental method is based on the ability to reduce dark purple DPPH in the presence of an antioxidant to a pale-yellow compound. A 1 mM solution of DPPH in 96% (*v*/*v*) ethyl alcohol was prepared by weighing 19.7 mg of DPPH dissolved in 50 mL of 96% EtOH. This solution was kept refrigerated in brown glass throughout the analysis. In parallel, a 2 mM solution of ascorbic acid in 96% ethanol was prepared by weighing 0.4 mg of ascorbic acid in one mL of the 96% EtOH solution. The ethanolic solution of ascorbic acid was considered the positive standard for the samples to be analysed.

According to a previously reported method, modified and developed by our research group [102], the antioxidant capacity of the eight samples of EOs was established using the DPPH free-radical-scavenging assay. Briefly, 0.5 mL of the sample solution to be analysed, 0.5 mL of the 1 mM DPPH alcohol solution, and 2 mL of the solvent (96% ethanol) are introduced into a 4 mL cuvette. The absorbance of the solutions is determined continuously at a wavelength of 516 nm for 1200 s using a T70 UV/VIS spectrophotometer (PG Instruments Ltd., Wibtoft, Lutterworth, United Kingdom). The same procedure is applied for the ascorbic acid solution: 0.5 mL of the 2 mM ascorbic acid solution is mixed with 0.5 mL of the 1 mM DPPH alcohol solution and 2 mL of the solvent (96% ethanol).

The antioxidant activity was calculated using the following formula:

(1) $$AOA\left(\%\right)=\left(\frac{A_{DPPH}-A_{EO}}{A_{DPPH}}\right)\times 100$$

where the variables are defined as follows:

AOA = is the antioxidant activity of the test samples analysed (%);

A_EO_ = is the absorbance of each EO test sample in the presence of the DPPH free radical measured at 516 nm;

A_DPPH_ = is the absorbance of the DPPH free radical measured at a wavelength of 516 nm without the EO test sample.

#### 4.2.2. Determination of Antimicrobial Activity

A. Disc-diffusion assay

The determination of antimicrobial activity was carried out both by the standardised disc-diffusion method (disc-diffusion susceptibility) and by the dilution method with the determination of the minimum inhibitory concentration (MIC) and the minimum bactericidal concentration (MBC) or minimum fungicidal concentration (MFC). The disc-diffusion method, being easier and cheaper to perform, has been used for screening the antimicrobial activity of EOs. The detailed work protocol is presented in Appendix B. According to the literature, a diameter ≥ 15 mm is considered highly susceptible [103].

B. Macro-dilution method

The macro-dilution technique allows for the determination of MIC and CMB/CMF.

The work principle applied was based on increasing dilutions of the antibacterial substance in tubes of liquid medium. Fixed amounts of the microbial culture are then added and incubated for 24 h at 37 °C, and the lowest concentration of antibacterial substance tested (essential oil) that does not allow bacterial growth is aimed for [104]. Also, the detailed working protocol can be seen in Appendix B.

C. Determination of CMB/CMF

Using a sterile disposable loop, 1 µL from each tube, including the control, was seeded on Columbia agar +5% sheep blood or Sabouraud with chloramphenicol and incubated 24 h at 37 °C, and then the highest dilution (lowest concentration) at which germs did not grow was read, representing the CMB/CMF [105].

#### 4.2.3. Determination of the Antitumour Activity

To determine the cytotoxic and anti-migratory activity of the EOs obtained from *Lamiaceae* species, two melanoma cell lines (A375 and B164A5) and a healthy keratinocyte cell line (HaCaT) were used. To achieve this objective, an MTT (3-(4,5-dimethylthiazol-2-yl)-2,5-diphenyltetrazolium bromide) analysis [97] was performed to determine cell viability after stimulation with the EOs, and a Scratch analysis [106,107] was performed to determine the anti-migratory effect of the EOs.

The A375 human melanoma cell line was purchased from the American Type Culture Collection (ATCC CRL-1619™) and B164A5 murine melanoma cells were purchased from Sigma-Aldrich (Munich, Germany). HaCaT human keratinocytes were provided by the Department of Dermatology, University of Debrecen, Hungary.

Cells were cultured in Dulbecco’s Modified Eagle Medium (DMEM 4.5 g/l glucose; Sigma-Aldrich, Taufkirchen, Germany), supplemented with 10% foetal bovine serum (FBS; Sigma-Aldrich, Taufkirchen, Germany) and antibiotics to avoid contamination (1% penicillin/streptomycin; Sigma-Aldrich, Taufkirchen, Germany). Cell growth was performed in a humidity-controlled atmosphere with 5% CO_2_ at 37 °C. For cell number determination, the Neubauer chamber was used in the presence of Trypan blue.

A. MTT assay

Cells were cultured in 96-well plates at a density of 1 × 104 cells/well and allowed to adhere to the well base overnight. Samples were applied in two concentrations (50 and 150 µg/mL) and incubated for 24 h with the cells. The MTT reagent—10 µL of 5 mg/mL MTT solution (Sigma-Aldrich, Budapest, Hungary) was added to each well (volume in the well was 100 µL). Intact mitochondrial reductase transformed and precipitated the MTT solution as blue crystals after a 3 h contact period.

Precipitated crystals were dissolved in 100 µL of the lysis solution (Sigma-Aldrich). Finally, samples were spectrophotometrically analysed at 570 using a microplate reader (xMark Microplate Spectrophotometer, Bio-Rad, Tokyo, Japan). The DMSO solution was used to prepare stock solutions for the samples tested [108].

B. Scratch assay

The migratory capacity of tumours (A375 and B164A5) and cells, respectively, was determined using the Scratch assay. The protocol was applied as previously described in the literature [109]. A number of 2 × 10^5^ cells/well were cultured in 12-well plates for 48 h before the experiment. A sterile pipette tip was used to draw a line in well-defined areas of the wells (at 80–90% confluence). Cells that detached as a result of the procedure were removed by washing with phosphate-buffered saline (PBS, Thermo Fisher Scientific, Cambridge, MA, USA). Cells were then stimulated with the lowest sample concentration (50 µg/mL). Images of cells in the culture were taken at the beginning of the experiment (0 h after stimulation) and at 12 and 24 h using the Olympus IX73 inverted microscope (Olympus, Tokyo, Japan) [107,109].

#### 4.2.4. Statistical Methods

Statistical calculations referring to the antimicrobial and antioxidant activity of the analysed EOs were performed using PAST 4.03 [110] and SAS Studio [111]. The evaluation of similarity and distances was performed using hierarchical clustering and the associated dendrogram. The assessment of differences between groups delimited by the application of a sample type was performed using ANOVA. Pairwise comparisons between groups were performed using Tukey’s pairwise test.

The results regarding the antitumour activity of the studied EOs were expressed as mean ± standard deviation. The comparison between groups was performed using One-way ANOVA, followed by Dunnett post-test. A *p*-value of ≤0.05 was considered to have statistical significance. Analyses were performed using GraphPad Prism 5 [112].

The Euclidean distances calculated for the measured diameters of the paired samples of the oils used were the basis of an analysis to determine similarities in their antimicrobial action. The formula used is $d=\sqrt{\sum_{i=1}^{n}{\left(x_{i}-y_{i}\right)}^{2}}$, where x_i_ represents the diameter values determined for the first plant sample type and y_i_ represents the diameter values determined for the second sample type.

## 5. Conclusions

The EOs from *Lamiaceae* species exhibit a wide array of pharmacological properties, including antimicrobial, antioxidant, anti-inflammatory, and anticancer activities. The rich chemical composition of these oils, characterized by various bioactive compounds, underpins their therapeutic potential. In the present study, we showed that the volatile oils obtained from plants belonging to the *Lamiaceae* family induced a significant antioxidant activity, the results being close to the value of ascorbic acid, which was used as a standard, especially in the case of *Lavandula* species. The results of this study confirm the traditional use of these species as antioxidants and suggest that the species cultivated in western Romania possess a high antioxidant capacity due to the polyphenols contained in the plants.

The EOs of *Lavandula angustifolia* Mill. (LA1), *Perovskia atripicifolia* Benth. (PA), *Lavandula hybrida* Balb. ex Ging (LH), *Lavandula angustifolia* Mill. (LA2), and *Salvia sclarea* L. (SS) were active only on Gram-positive cocci and fungi. The other EOs, *Mentha smithiana* L. (MS) and *Mentha x piperita* L. (MP), had inhibitory activity on all the reference strains tested. The EO of *Mentha smithiana* L. (MS) shows antibacterial and antifungal activity on all strains tested, with a maximum zone of inhibition on the fungi *C. albicans* and *C. parapsilosis*. For the EO of *Salvia officinalis* L. (SO), no antibacterial activity was recorded, while the EO of *Perovskia atriplicifolia* Benth. (PA) was active only on Gram-positive cocci and fungi.

From the perspective of cytotoxic activity, the obtained results indicated that the samples with the highest efficiency were *Salvia officinalis* L. (SO) and *Perovskia atripicifolia* Benth. (PA). The analysed EOs show a dose-dependent cytotoxic effect. These samples do not show selectivity against tumour cells because, at high doses, they also reduced keratinocyte viability.

In terms of anti-migratory capacity, the data obtained indicate that many samples reduced the migratory capacity of melanoma cell lines. The comparative evaluation of the results obtained on the human melanoma line and human keratinocytes indicated that LA1 and MP had the most potent effect by inhibiting the migration ability of tumour cells and stimulating HaCaT migration. The EO samples showed increased selectivity; they stimulated the migration of healthy cells and inhibited the migratory capacity of tumour cells.

EOs are bio-compounds with many potential applications and uses in pharmaceutics. This direction shall be deepened due to the high demand for effective and affordable therapeutical solutions. Future research should focus on elucidating the mechanisms of action of these oils and exploring their applications in both clinical and industrial settings.

## Figures and Tables

**Table 1:** The antioxidant capacity [%] of the EOs obtained from cultivated *Lamiaceae* medicinal species, as compared with standard (ascorbic acid), and the corresponding IC_50_ values, respectively.

Sample No.	Sample Code	Antioxidant Capacity [%]	IC_50_ ± SD [μg/mL]
1	LA1	88.85 ± 0.024	15.84 ± 0.92
2	LA2	90.90 ± 0.002	13.28 ± 0.67
3	SO	55.56 ± 0.187	94.73 ± 1.18
4	LH	83.81 ± 0.004	19.39 ± 0.98
5	SS	52.05 ± 0.079	109.28 ± 1.34
6	MS	70.02 ± 0.117	65.78 ± 1.21
7	PA	38.81 ± 0.041	186.84 ± 1.56
8	MP	89.18 ± 0.003	45.75 ± 1.06
9	Ascorbic acid	95.92 ± 0.026	0.7 ± 0.05

**Table 2:** The diameters of the inhibition zones obtained by the disc-diffusion method (mm).

EO	*K. pneumoniae*	*S. flexneri*	*S. enterica*	*E. coli*	*P. aeruginosa*	*S. aureus*	*E. faecalis*	*C. albicans*	*C. parapsilosis*
MS	21	20	22	22	15	21	22	33	33
SO	6	9	9	9	6	10	9	10	10
LA1	13	14	10	14	6	20	19	30	30
PA	6	6	6	10	6	20	16	20	19
LH	6	6	9	10	6	21	20	20	21
MP	25	20	20	26	21	26	24	20	20
LA2	10	11	9	9	6	16	15	19	18
SS	6	6	6	6	6	22	20	20	20

**Table 3:** Matrix of the Euclidean distances (Source: authors’ own calculations using PAST 4.03).

EO	MS	SO	LA1	PA	LH	MP	LA2	SS
MS	0.00							
SO	45.97	0.00						
LA1	20.42	33.17	0.00					
PA	36.11	18.68	19.36	0.00				
LH	33.65	21.75	17.69	5.48	0.00			
MP	20.95	42.40	30.17	36.54	34.66	0.00		
LA2	33.41	15.39	18.47	8.37	10.10	33.57	0.00	
SS	36.84	22.18	19.95	6.08	5.20	37.55	11.18	0.00

**Table 4:** MIC and MBC/MFC values obtained by the macro-dilution method.

EO	*K. pneumoniae*		*S. flexneri*		*S. enterica*		*E. coli*		*P. aeruginosa*		*S. aureus*		*E. faecalis*		*C. albicans*		*C. parapsilosis*	
	MIC	MBC	MIC	MBC	MIC	MBC	MIC	MBC	MIC	MBC	MIC	MBC	MIC	MBC	MIC	MBC	MIC	MBC
MS	10	20	10	20	10	20	10	20	20	40	10	10	10	10	5	5	5	5
LA1											10	20	10	20	5	5	5	5
PA											10	20	10	20	10	10	10	10
LH											10	20	10	20	10	10	10	10
MP	10	20	10	20	10	20	10	20	20	40	5	10	10	10	10	10	10	10
LA2											10	20	20	20	10	10	10	10
SS											20	20	20	20	10	10	10	10

**Table 5:** Average drying efficiency and the amount of essential oil obtained from the studied species.

Sample Code	Fresh *herba* (g)	Dry *herba* (g)	Drying Efficiency (%)	EO from Sample (mL)	EO ml/100 g Dry *herba*
LA1	3450	1100	31.88	15	1.36
LA2	2968	1012	29.33	18	1.78
SO	2843	987	33.25	4.6	0.47
LH	3526	1188	33.69	20.1	1.69
SS	2487	795	31.96	10.1	1.27
MS	2507	890	35.5	5.5	0.61
PA	2717	918	33.78	8.3	0.9
MP	11700	3630	31.02	40.5	1.11

**Table 6:** Reference strains.

Microbial Species	ATCC	Manufacturer
*Salmonella enterica* serotype *typhimurium*	14028	Thermo Scientific (Waltham, MA, USA)
*Shigella flexneri* serotype 2b	12022	
*Enterococcus faecalis*	51299	
*Escherichia coli*	25922	
*Klebsiella pneumoniae*	700603	
*Pseudomonas aeruginosa*	27853	
*Staphylococcus aureus*	25923	
*Candida albicans*	10231	
*Candida parapsilosis*	22019	

## Data Availability

Data are available from the corresponding authors.

## Appendix A

**Table A1:** Chemical composition of the essential oil of *Lavandula angustifolia* Mill. (LA1).

Chemical Compound	%	RT (min)
Eucalyptol	2.144	
Trans-Beta-Ocimene	5.976	10.836
cis-beta-Ocimene	2.477	14.876
Linalool	22.11	17.589
endo-Borneol	2.23	18.043
Terpinen-4-ol	3.811	18.59
Alpha-Terpineol	2.13	21.202
Linalyl acetate	20.384	22.22
Lavandulyl acetate	7.599	26.35
Carvacrol	3.333	27.255
Diphenhydramine	7.31	28.742
cis-beta-Farnesene	3.86	31.771
tau-Cadinol	2.473	

**Table A2:** Chemical composition of the essential oil of *Lavandula angustifolia* Mill. (LA2).

Chemical Compound	%	RT (min)
Alpha-Thujene	0.12	5.656
Alpha-Pinene	0.38	5.893
Camphene	0.33	6.379
Sabinene	0.6	7.487
Beta-Pinene	0.51	8.075
n-Hexyl acetate	0.2	8.908
3-Carene	0.26	9.139
p-Cymene	0.13	9.643
Benzene, 1-methyl-3-(1-methylethyl)-	0.84	9.861
Eucalyptol	1.45	10.126
D-Limonene	1.02	10.193
trans-beta-Ocimene	2.18	10.819
1,3,6-Octatriene, 3,7-dimethyl-, (Z)-	0.85	11.577
Sabinene hydrate	0.49	13.177
Linalool	30.15	15.322
(+)-2-Bornanone	1.57	16.321
endo-Borneol	2.46	18.258
(−)-4-Terpineol	7.19	18.47
Alpha-Terpineol	1.83	19.243
Borneol, acetate	0.24	19.983
cis-(−)-1,2-Epoxy-p-menth-8-ene	0.16	20.228
Linalyl acetate	23.71	21.207
(Z)-Geraniol	0.81	21.605
Bicyclo[2.2.1]heptan-2-ol, 1,7,7-trimethyl-, acetate, (1S-endo)-	0.39	22.004
Lavandulol acetate	6.21	22.251
Hexyl tiglate	0.07	23.491
(R)-lavandulyl (R)-2-methylbutanoate	0.59	24.482
cis-Geranyl acetate	0.9	25.03
Hexanoic acid, hexyl ester	0.23	25.15
(−)-beta-Bourbonene	0.09	25.35
Sesquithujene	0.1	25.497
Beta-Curcumene	0.05	25.934
Alpha-Cedrene	0.1	26.183
Bicyclo[5.2.0]nonane, 2-methylene-4,8,8-trimethyl-4-vinyl-	4.79	26.361
cis-alpha-Bergamotene	0.2	26.768
Aromandendrene	0.12	27.005
Bicyclo[7.2.0]undec-4-ene, 4,11,11-trimethyl-8-methylene-,[1R-(1R*,4Z,9S*)]-	2.54	27.26
Hexadecane	0.21	27.59
Germacrene D	0.19	28.001
(R)-lavandulyl (R)-2-methylbutanoate	0.05	28.51
Alpha-Guaiene	0.12	28.596
Naphthalene, 1,2,3,4,4a,5,6,8a-octahydro-7-methyl-4-methylene-1-(1-methylethyl)-, (1.alpha.,4a.beta.,8a.alpha.)-	1.07	28.853
delta-Cadinene	0.18	29.003
Bicyclo[2.2.1]hept-2-ene, 1,7,7-trimethyl-	0.07	29.165
1-Methyl-6-(3-methylbuta-1,3-dienyl)-7-oxabicyclo[4.1.0]heptane	0.1	29.792
Spiro[tricyclo[3.3.1.1(3,7)]decane-2,2′-oxetan]-4′-one, 3′-methylene-	0.16	30.155
Caryophyllene oxide	2.63	30.589
(1R,3E,7E,11R)-1,5,5,8-Tetramethyl-12-oxabicyclo[9.1.0]dodeca-3,7-diene	0.1	31.239
Epicubenol	0.12	31.488
tau-Cadinol	1.06	32.165
Muurol-5-en-4-one <cis-14-nor->	0.08	33.115

**Table A3:** Chemical composition of the essential oil of *Salvia officinalis* L. (SO).

Chemical Compound	%	RT (min)
5-Undecene, 7-ethenyl-	0.32	3.832
3-Decyne	0.07	4.013
Alpha-Thujene	0.22	5.663
1R-alpha-Pinene	2.59	5.908
Camphene	2.17	6.385
Sabinen	9	7.488
Beta-Pinene	0.34	8.083
Eucalyptol	1.04	9.913
D-Limonene	15.04	10.209
trans-beta-Ocimene	0.15	10.843
Sabinene hydrate	0.12	13.119
Beta-Thujone	16.84	14.713
Alpha-Thujone	8.81	15.247
(+)-2-Bornanone	3.83	16.317
trans-Sabinol	0.2	16.87
L-Pinocarveol	0.37	17.127
L-Borneol	6.8	18.259
trans-Ocimenol	0.37	19.211
(−)-Myrtenol	0.19	19.523
Pentanoic acid, 4-hexen-1-yl ester	0.17	20.305
Linalyl acetate	0.05	21.075
L-bornyl acetate	0.73	21.995
Sabinyl isobutanoate	0.06	22.261
Alpha-Cubebene	0.07	24.308
Alpha-ylangene	0.04	24.977
Alpha-Copaene	0.12	25.114
(−)-beta-Bourbonene	0.09	25.347
Bicyclo[5.2.0]nonane, 2-methylene-4,8,8-trimethyl-4-vinyl-	0.07	25.96
Isocaryophyllene	6.33	26.375
Humulene	5.33	27.321
Gamma-Muurolene	0.24	27.859
(+)-Ledene	0.06	28.376
Alpha-Cadinene	0.06	28.51
Naphthalene, 1,2,3,4,4a,5,6,8a-octahydro-7-methyl-4-methylene-1-(1-methylethyl)-, (1alpha,4a.beta.,8a.alpha.)-	0.11	28.853
Delta-Cadinene	0.18	29.033
1-Methyl-6-(3-methylbuta-1,3-dienyl)-7-oxabicyclo[4.1.0]heptane	0.27	29.796
Caryophyllene oxide	3.61	30.599
Viridiflorol	7.4	31.057
(1R,3E,7E,11R)-1,5,5,8-Tetramethyl-12-oxabicyclo[9.1.0]dodeca-3,7-diene	2.6	31.262
Copalol	0.2	31.751
Caryophylla-4(12),8(13)-dien-5alpha-ol	0.2	32.188
cis-alpha-Bisabolene	0.06	35.543
Cyclopentadecanone	0.15	36.394
Butyl 6,9,12,15-octadecatetraenoate	0.37	36.737
Isopimara-9(11),15-diene	0.2	38.46
n-Hexadecanoic acid	0.15	40.968
Epimanool	2.46	42.17
(+)-Valencene	0.15	42.631

**Table A4:** Chemical composition of the essential oil of *Lavandula hybrida* Balb. ex Ging (LH).

Chemical Compound	%	RT (min)
Alpha-Pinene	1.14	5.983
Camphene	0.45	6.43
Beta-Ocimene	1.85	7.491
Beta-Pinene	1.07	8.092
Eucalyptol	17.84	10.116
Limonene	2.01	10.225
trans-beta-Ocimene	5.42	10.748
Beta-Ocimene	1.91	11.487
Gamma-Terpinene	0.47	13.847
Linalool	35.86	14.633
(+)-2-Bornanone	6.06	15.902
endo-Borneol	6.8	17.478
Terpinen-4-ol	3.62	17.906
Alpha-Terpineol	1.98	18.473
Linalyl acetate	5.41	20.977
Lavandulol acetate	0.86	22.084
Caryophyllene	1.04	26.247
cis-beta-Farnesene	4.19	27.192
Germacrene D	0.74	27.859
(R)-lavandulyl (R)-2-methylbutanoate	0.42	28.435
Alpha-Bisabolol	0.86	32.678

**Table A5:** Chemical composition of the essential oil of *Salvia sclarea* L. (SS).

Chemical Compound	%	RT (min)
Beta-Pinene	0.52	8.102
Beta-Ocimene	0.65	11.484
Linalool	10.12	14.553
Alpha-Terpineol	1.15	18.483
Linalyl formate	1.01	19.463
Linalyl acetate	69.41	21.099
Neryl acetate	0.69	24.32
Lavandulol acetate	1.09	24.856
Alpha-Cubebene	0.71	25.074
Aromandendrene	3.23	26.252
Germacrene D	8.61	27.87
(1S,2E,6E,10R)-3,7,11,11-Tetramethylbicyclo[8.1.0]undeca-2,6-diene	1.56	28.271
Alpha-Farnesene	0.43	28.503
Alloaromadendrene	0.82	30.819

**Table A6:** Chemical composition of the essential oil of Mentha *smithiana* L. (MS).

Chemical Compound	%	RT (min)
Camphenol, 6-	0.048	9.498
Tricyclenne	0.006	6.942
A-phellandrene	0.03	9.425
A-pinene	1.009	5.983
Camphene	0.223	6.43
Sabinene	0.912	8.200
B-pinene	1.515	7.747
Limonene	14.18	10.225
Eucalyptol	0.56	9.913
1,3,6 octatriene 3,7 dimethyl (Z) (beta cis ocimene)	0.071	11.577
A-terpinene	0.029	6.726
P-mentha-1,4(8) diene	0.084	10.98
Linalool	0.336	15.322
P-menth-1-en-8-ol	0.064	18.573
Geraniol acetate	0.256	23.70
B-caryophilene	2.12	26.824
B-farnesene	0.763	26.33

**Table A7:** Chemical composition of the essential oil of *Perovskia atriplicifolia* Benth. (PA).

Chemical Compound	%	RT (min)
Phellandrene	0.14	7.85
2-hexanal	0.03	4.97
Trans-sabinene hydrate	0.19	12.523
borneol	4.79	28.833
camphene	1.45	6.707
sabinene	1.02	6.91
P-cymene	0.32	9.748
Beta-myrcene	0.53	6,98
1,8-cineole	5.85	10.105
3-carene	7,56	9.226
Alpha-terpinene	0.25	6.726
o-cymene	0.38	10.30
Limonene	0.49	10.250
Cis-ocimene	0.75	10.82
Beta-ocimeneY	0.57	8.59
y-terpinene	0.46	9.78
Dehydro-p-cymene	0.03	11.09
Alpha-terpinolene	0.04	10.98
Cis--sabinene hydrate	0.06	10.20
linalool	5.21	19.548
Iso-amyl isovalerate	0.03	11.60
camphor	2.12	18.787
4-terpineol	0.42	17.93
Cumyl alcohol	0.04	2.429
Alfa-terpinol	0.65	18.573
methylcyclohexane	0.07	2.61
calarene	0.98	19.95
valeranone	2.34	35.74
Alpha-thujene	0.07	5,40
Bornyl formate	0.44	17.36
Beta-Pinene	0.98	7.04
Alpha-Pinene	4.30	5.85

**Table A8:** Chemical composition of the essential oil of *Mentha x piperita* L. (MP).

Chemical Compound	%	RT (min)
Alpha-Pinene	0.93	5.994
3-Octanone	0.75	7.324
Beta-Pinene	1.38	7.51
Beta-Myrcene	0.7	8.114
Eucalyptol	10.26	10.129
D-Limonene	9.7	10.272
Trans-beta-Ocimene	0.94	10.755
Bicyclo[3.1.0]hexan-2-ol, 2-methyl-5-(1-methylethyl)-, (1.alpha.,2.beta.,5.alpha.)-	4.43	12.57
Alpha-Terpineol	0.31	18.508
8-p-Menthen-2-ol	5.58	18.749
D-Carvone	42.28	20.207
Borane carbonyl	0.68	20.495
8-p-Menthen-2-yl, acetate, trans	1.28	23.256
2-Cyclohexen-1-ol, 2-methyl-5-(1-methylethenyl)-, acetate, (1R-cis)-	0.57	24.306
Jasmone	0.48	25.06
Beta-Bourbonene	1.83	25.298
Beta-Elemene	0.85	25.485
Caryophyllene	4.94	26.269
Isogermacrene D	0.17	26.91
Bicyclosesquiphellandrene	0.34	26.962
Beta-copaene	0.68	27.395
Germacrene D	8.47	27.887
Gamma-Elemene	1.65	28.286
Alpha-Selinene	0.8	28.516

## Appendix B

Determination of antimicrobial activity (detailed)

A. Disc-diffusion assay

The determination of antimicrobial activity was carried out both by the standardised disc-diffusion method (disc-diffusion susceptibility) and by the dilution method, with the determination of the minimum inhibitory concentration (MIC) and the minimum bactericidal concentration (MBC) or minimum fungicidal concentration (MFC).

The disc-diffusion method, being easier and cheaper to perform, has been used for screening the antimicrobial activity of EOs.

Principle of the method used: The antimicrobial substance (essential oil) is deposited on the surface of an agar culture medium, which has been pre-seeded with the test bacteria. Two phenomena occur simultaneously: the diffusion of the oil into the medium and multiplication of the micro-organism. In areas where the oil achieves concentrations higher than the MIC, bacterial growth no longer occurs. The circumference of the inhibition zone is established from the first hours of incubation as the geometric location of the points where the oil has reached the MIC at the critical time of culture. Thus, the diameter of the inhibition zone varies inversely with the MIC.

The composition of the culture medium, its pH, inoculum density, stability and diffusion of the test substance (oil), and incubation time and temperature, are all variables that influence the results of the disc-diffusion method.

Materials needed:

1. Reference strains were seeded on Columbia agar +5% sheep blood and Sabouraud with fungal chloramphenicol, respectively, with 24 h thermostatting at 37 °C. The inoculum density, i.e., the number of bacteria brought into contact with the tested oil, is an important element and condition for the reproducibility of the results. According to the CLSI [Clinical and Laboratory Standards Institute] standard, a microbial suspension in sterile saline equivalent to 0.5 Mc Farland (108 CFU/mL) is prepared [103].
2. For the culture medium, we used Mueller-Hinton agar (bioMerieux, Marcy-l’Étoile, France), which is recommended by CLSI. For the Candida strains, we used Mueller-Hinton medium supplemented with methylene blue. The sterility control of the media consisted of incubating a plate from the batch used for 24 h at 37 °C.
3. An unimpregnated microcompressed blank, 6 mm in diameter (BioMaxima, Lublin, Poland), was used.
4. Other materials used include sterile saline, cotton wool pads on wooden rods, and tweezers for the deposition of microcompresses.

The technique of the disc-diffusion antibiogram is as follows: standardized bacterial suspension was obtained by suspending colonies from the 24 h culture on Columbia agar +5% sheep blood and Sabouraud medium with chloramphenicol for *Candida* strains, respectively, in sterile saline.

Seeding was carried out with a sterile cotton swab, which was dipped into the suspension; excess was removed by pressing against the walls of the tube and passing over the surface of the medium in three directions until the entire surface was covered, with a circular marginal streak being made to obtain a uniform inoculum.

After inoculation, the plates were left for 5–10 min at room temperature to facilitate inoculum uptake into the medium, after which the microcompresses were deposited with a minimum distance of 25 mm between discs and 15 mm from the edge of the Petri dish. From the undiluted test oils, 10 µL per tablet was deposited.

For each oil, a single plate was used, on which the micro-tablet with the oil and two control micro-tablets were deposited. The positive control consisted of a Gentamicin micro-tablet of 10 µg and 120 µg, respectively, for the *Enterococcus faecalis* strain. For *Candida* strains, the positive control was a 10 µg Fluconazole micro-tablet. An unimpregnated micro-tablet was used as a negative control. The plates thus prepared were incubated for 24 h at 35 ± 2 °C.

The interpretation of results was as follows: after thermostatting, readings were taken by measuring the diameters of the inhibition zones with a ruler. According to the literature, a diameter ≥ 15 mm is considered highly susceptible [104].

For quality control, all tests were performed in duplicate.

B. Macro-dilution method

The macro-dilution technique allows for the determination of MIC and CMB/CMF.

The work principle is as follows: increasing dilutions of the antibacterial substance are made in tubes of the liquid medium. Fixed amounts of the microbial culture are then added and incubated for 24 h at 37 °C, and the lowest concentration of the antibacterial substance tested (essential oil) that does not allow bacterial growth is aimed for [103].

The materials required are as follows: sterile test tubes, liquid culture medium (Mueller-Hinton broth), test oils, 0.5 Mc Farland microbial suspension from reference strains, pipettes, and a thermostat.

The working method is as follows: An inoculum of approximately 5 × 10^5^ CFU/mL is prepared from the 0.5 Mc Farland bacterial suspension. This can be achieved by diluting the 0.5 McFarland suspension to a ratio of 1:150, resulting in a suspension of 10^6^ CFU/mL. Further dilution at a ratio of 1:2 will bring the final inoculum to 5 × 10^5^ CFU/mL [103].

Testing was carried out in five test tubes as follows: 0.5 mL of the 5 × 10^5^ CFU/mL bacterial suspension was pipetted into each test tube; then, 0.4 mL of Mueller-Hinton broth and 0.1 mL of the essential oil dilution was obtained in DMSO (1/2, 1/4, 1/8, 1/16, 1/32), resulting in a volume of 1 mL, which was homogenised. For each strain tested, a positive control containing 0.5 mL of the bacterial suspension, 0.4 mL of Mueller-Hinton broth, and 0.1 mL of DMSO was used to check the growth of the strain. The negative control contained 0.1 mL of oil and 0.4 mL of Mueller-Hinton broth +0.5 mL of DMSO.

For the interpretation, first, the control is read, which should be turbid (if the germ has grown). If there is no bacterial growth, the broth in the control tube should remain clear, invalidating the test. If the blank is adequate, the reaction has been performed correctly and the reading is taken from the tubes in which the medium has remained unchanged (the germ has not grown because it is inhibited by the oil). The lowest concentration at which no germ growth has occurred is the CMI.

C. Determination of CMB/CMF:

Using a sterile disposable loop, 1 µL from each tube, including the control, was seeded on Columbia agar +5% sheep blood or Sabouraud with chloramphenicol and incubated 24 h at 37 °C; then, the highest dilution (lowest concentration) at which germs did not grow was read, representing the CMB/CMF [105].
